# Assessment of Acceptability and Determinants of Uptake and Schedule Completion of Human Papillomavirus (HPV) Vaccine by 25 to 45 Years Old Women in Slovenia

**DOI:** 10.3390/vaccines11020423

**Published:** 2023-02-12

**Authors:** Jana Mlakar, Anja Oštrbenk Valenčak, Jožefa Kežar, Lara Beseničar-Pregelj, Mario Poljak

**Affiliations:** 1 Institute of Microbiology and Immunology, Faculty of Medicine, University of Ljubljana, Zaloška 4, SI-1000 Ljubljana, Slovenia; 2 Litija Community Health Center, Partizanska pot 8a, SI-1270 Litija, Slovenia; 3 Nova Gorica Community Health Center, Rejčeva ulica 4, SI-5000 Nova Gorica, Slovenia

**Keywords:** HPV vaccination, acceptability, adult women, Slovenia

## Abstract

HPV immunization programs are mainly focused on girls and boys, but adult women and men could also benefit from vaccination. A multinational CoheaHr-WP4 study investigated the acceptability of HPV vaccination among 25–45 years old women. A total of 607 women from Slovenia participated in the study, and 49.6% (301/607) agreed with HPV vaccination, with a significant difference (*p* < 0.0001) between the two centers. Non-vaccinated women had a higher education (*p* = 0.0068) and were more frequently in a committed relationship or married (*p* = 0.01). The most trusted source of medical and vaccination information was healthcare providers (55.2%). The main reasons for vaccine acceptance were protection against HPV-related disease (93.4%), severity of preventable diseases (82.7%), HPV vaccine safety (66.8%), free HPV vaccine availability (62.8%), and the existence of vaccination recommendations (55.5%). The main reasons for refusing vaccination were the need for additional vaccine-related information (31.4%) and vaccine safety concerns (29.4%). To increase vaccine coverage, information about the benefits and safety of HPV vaccination must be widely disseminated to all health professionals and the general public. We are convinced that the knowledge obtained in this study can be reliably applied to other countries in the region that lack such information and have a very high cervical cancer burden.

## 1. Introduction

Cervical cancer, which is the fourth most common cancer in women worldwide and led to more than 340,000 deaths in 2020, is caused by persistent human papillomavirus (HPV) infection [1]. Two revolutionary advances have recently opened new prospects for the elimination of cervical cancer: prophylactic HPV vaccination as a primary prevention tool and HPV testing as a secondary prevention (screening) tool [2]. These two prevention options complement and enhance established cervical cancer prevention programs and provide robust solutions, especially in low-resource settings with a high burden of disease and without optimally functioning screening programs. Adequately combined, they have the potential to dramatically reduce cervical cancer incidence and mortality; in fact, no other neoplastic disease affecting humans can currently rival the magnitude of this potential [2].

The first HPV vaccine—the quadrivalent HPV6/11/16/18 Gardasil vaccine (Merck & Co, Rahway, NJ, USA)—was licensed in Europe in 2006, followed by the bivalent HPV16/18 Cervarix vaccine (GSK Biologicals, Rixensart, Belgium) in 2007, and finally by the nonavalent HPV6/11/16/18/31/33/45/52/58 Gardasil 9 vaccine (Sanofi Pasteur MSD SNC, Lyon, France) in 2015. Since 2006, the great majority of European countries have implemented HPV vaccination in their national vaccination programs [3], with recommendations mainly for the vaccination of both preadolescent girls and boys, accompanied by catchup programs for older cohorts in selected countries. Countries that reached high HPV vaccination coverage across targeted multi-age cohorts achieved a dramatic decrease in the incidence of anogenital warts [4], precancerous lesions of the cervix [5,6], anus [7], vagina [7], and vulva [7], and recently also cervical cancer [6,8,9,10] and laryngeal papillomas [11].

In Slovenia, a central European country with a population of 2.1 million, the crude incidence rate of cervical cancer was between 8.3 and 12.4 per 100,000 women between 2017 and 2020, and it has significantly decreased from 20.7 per 100,000 women in 2003 when the National Cervical Cancer Screening Program (NP ZORA) was implemented [12,13]. This organized national program targets women between ages of 20 and 64 who receive conventional cytology (Pap smear) screening within a primary health care network of gynecologists every three years, after two negative screening Pap smears in 12 months upon entry into the program. The screening coverage rate was 71.7% between 2018 and 2021 with some age- and region-related differences [13]. In addition to a well-functioning cervical cancer screening program, vaccination against HPV was gradually introduced in Slovenia in 2007 and was finally implemented in the national HPV vaccination program in September 2009. In the national program, HPV vaccination was initially offered free of charge to girls 11 to 12 years old in the sixth grade of primary school only and later also to all those that had missed their initial appointments. By 2014, girls had received three doses of the quadrivalent HPV vaccine, and in 2014 vaccination with two doses of the quadrivalent HPV vaccine was implemented. Since the 2016/17 school year, the nonavalent HPV vaccine has been used for girls in the sixth grade of primary school. The national vaccination coverage for three/two doses of HPV quadrivalent/nonavalent vaccine among eligible girls gradually increased and reached 59.3% in the 2018/19 school year, with important differences between geographical regions [14]. Traditionally, the lowest vaccination coverage rates among eligible girls were recorded in the Ljubljana (capital) region (around 36%) and the highest in the Ravne region (around 85%). In 2019, the Slovenian national HPV vaccine program was expanded to boys 11 to 12 years old, and coverage for two doses of HPV nonavalent vaccine reached 14.73% in the 2021/22 school year among eligible boys [15].

In addition to girls and boys targeted in the great majority of national vaccination programs worldwide, adult women and men could also benefit from HPV vaccination [16]. Women aged 25 to 45 years account for approximately one third of those eligible for cervical cancer screening. The highest incidence and cross-sectional prevalence of HPV infection are around age 20, the peak incidence/detection of grade 3 cervical intraepithelial neoplasia is around age 30, and the peak incidence of cervical cancer is in the 40s [17]. HPV16 and HPV18 are the most important cancer-related genotypes causing 70% of all cervical cancers. A study assessing the burden of high-risk HPV cervical infection in Slovenia determined an overall point prevalence of 12.9%, 3.5% for HPV16, and 1.0% for HPV18 in women aged 20 to 64 years [18]. However, among Slovenian women aged 30 to 39 years, the seroprevalence for high-risk HPV genotypes (reflecting cumulative or lifetime incidence) was 64.0%, whereas it was 35.2% for the HPV16 or HPV18 genotypes [19]. The HPV-FASTER strategy, which aims to significantly accelerate global cervical cancer elimination, suggests HPV screening, treatment if needed, and HPV vaccination of adult women at a screening visit [20]. This is founded on the results of two phase III trials that proved good vaccine efficacy in adult women that were HPV DNA-negative at the time of vaccination. However, there is still a limited understanding of the effectiveness of HPV vaccination in adult women and its impact on reducing cervical cancer incidence and mortality and the reduction of screening needs [20,21]. In addition, there are only scant data on acceptability of the HPV vaccine among adult women and men and on vaccination coverage among non-targeted adult women and men in Europe. To fill this knowledge gap, a multinational, open-label COHEAHR-WP4 study conducted between April 2016 and May 2018 enrolled a total of 3646 women 25 to 45 years old attending opportunistic or population-based cervical cancer screening in nine European countries—Belgium, Denmark, Finland, France, Germany, Slovenia, Spain, Sweden, and the United Kingdom—and offered them HPV vaccination at no cost [22]. Overall, 2151 (66.9%) were vaccinated with the first dose, and 1958 (60.9%) received the full vaccination course [22]. Here we present the results obtained for 607 Slovenian women that participated in the COHEAHR-WP4 multinational study. We aimed to determine the acceptability of HPV vaccination by adult women in Slovenia, to identify determinants of uptake and completion of the vaccination schedule, and to closely monitor potential adverse vaccine effects in the target population. Due to similarity with other countries in central and eastern Europe, where such information is lacking, we are convinced that knowledge obtained in this study can be safely transferred to other countries in the region, some of which have an extremely high burden of cervical cancer.

## 2. Materials and Methods

The COHEAHR-WP4 study was part of the COHEAHR project funded under the European Commission FP7 Framework Health 2013 Innovation 1 initiative (grant no. 603019; “Comparing Health Services Interventions for the Prevention of HPV-Related Cancer Project”; EudraCT no. 2014-003177-42). Participating countries obtained national ethical and regulatory approvals. In Slovenia, the study was approved by the National Medical Ethics Committee (consent no. 0120-657/2015-2-KME 81/12/15) and by the Agency for Medicinal Products and Medical Devices of the Republic of Slovenia (consent no. 1050-17/2016-3).

For the Slovenian part of the COHEAHR-WP4 study, two gynecologists from different regions—Litija (Center 1) and Nova Gorica (Center 2)—recruited women to participate in the study between August 2016 and October 2018. Both gynecologists consequently invited women 25 to 45 years old with no previous history of HPV vaccine administration that attended the routine organized national screening program until they reached the target number of participants. As in other countries participating in the COHEAHR-WP4 study, the same core protocol and data collection forms were used. Before the COHEAHR-WP4 study launch, the questionnaire was validated in a small pilot study. The women were given a brochure with information about cervical cancer, HPV, the HPV vaccine, and the aims of the study, as well as a detailed summary of the study protocol, and they were invited to participate in the first part of the study (completing a detailed anonymous questionnaire consisting of 20 questions; Supplement S1) and/or in the second part of the study (HPV vaccination). All women that agreed to participate in the study signed a written informed consent form. The women that opted for HPV vaccination received the first HPV vaccine dose, preferably during their initial visit. If they needed additional time to decide about HPV vaccination, the first vaccine dose was administered at their next visit. If any women refused to participate in the study, they were asked to provide reason(s) for declining.

The women were vaccinated with Gardasil 9 (Sanofi Pasteur MSD, Lyon, France) targeting nine HPV genotypes: HPV6/11/16/18/31/33/45/52/58. Exclusion criteria for vaccination were the following: known history of severe allergic reactions or hypersensitivity to any of the vaccine components; known history of immune-related disease; current acute severe febrile illness; administration of immunoglobulin or blood-derived products within six months before the planned first dose of HPV vaccine; pregnancy or planning to become pregnant in the following 12 months; and women that had undergone hysterectomy.

Study data were collected and managed using REDCap (Research Electronic Data Capture), a secure web-based application designed to support data collection for research studies. It provides (i) an intuitive interface for validated data entry, (ii) audit trails for tracking data manipulation and export procedures, (iii) automated export procedures for seamless data downloads into common statistical packages, and (iv) procedures for importing data from external sources [23].

Statistical significance tests were performed using the mid-P exact test for categorical data and the *t*-test for continuous data available in the open-source program OpenEpi 3.01 (http://www.openepi.com/Menu/OE_Menu.htm (accessed on 13 May 2019). Values *p* ≤ 0.05 were considered statistically significant.

## 3. Results

A total of 607 women—363 (59.8%) at Center 1 and 244 (40.2%) at Center 2—met the inclusion criteria and decided to participate in the study. Of these, 306 (50.4%) participated only in the first part of the study (the anonymous questionnaire) and 301 (49.6%) participated in both study parts (the anonymous questionnaire and HPV vaccination; Table 1). Of the 301 participants vaccinated with the first dose of HPV vaccine, 95.7% (288/301) received a full three-dose HPV vaccination. Participating Slovenian women represented 16.6% of the total study population of the COHEAHR-WP4 multinational study and were the only women enrolled in this project from outside western Europe [22].

As shown in Table 1, the mean age of the Slovenian participants was 35.5 ± 2.8 years. Of all participants, 43.5% (264/607) had completed secondary school or high school, whereas 42.8% (260/607), 10.9% (66/607), and 2.8% (17/607) had completed university, another educational level, and elementary school, respectively. A statistically significant difference (*p* = 0.0068) was found in the educational level between the vaccinated and non-vaccinated, with the non-vaccinated having a higher educational level. A statistically significant difference was also found in marital status, with more non-vaccinated women being in a committed relationship or married. Of all participants, 80.6% (489/607) were in a committed relationship or married, whereas 17.1% (104/607) were single or divorced, and 0.5% (3/607) were widowed. According to the questionnaire data, 3.3% (20/607) of participants had already had anogenital warts. The great majority (84.0%; 510/607) of the participants enrolled had previously attended cervical cancer screening, 76.4% (464/607) attended screening regularly, and 72.2% (438/607) had had more than two cervical cancer screenings in their lifetime. Significantly more non-vaccinated individuals had previously participated in cervical cancer screening (*p* = 0.0077). Reasons for not attending cervical cancer screening regularly included lack of time (38.2%; 13/34), discomfort with the procedure (23.5%; 8/34), misinformation or lack of information on the need for periodic cervical screening (5.9%; 2/34), economic reasons (including work leave and travel costs) (2.9%; 1/34), and other reasons (20.6%; 7/34). Only 1.6% (9/575) of participants had a history of medical problems linked with any previous (non-HPV) vaccinations, with no significant differences observed between HPV vaccinated versus HPV non-vaccinated in this study. Of all participants, 78.4% (476/607) of participants have correct knowledge that cervical cytology or a Pap test is the main tool used in cervical cancer screening in Slovenia, and 27.5% (167/607) wrongly thought that HPV testing is the main screening tool in Slovenia. Interestingly, significantly more HPV vaccinated versus HPV non-vaccinated in this study thought that HPV is used as the main cervical cancer screening tool in Slovenia (*p* = 0.0001).

**Figure 1:**
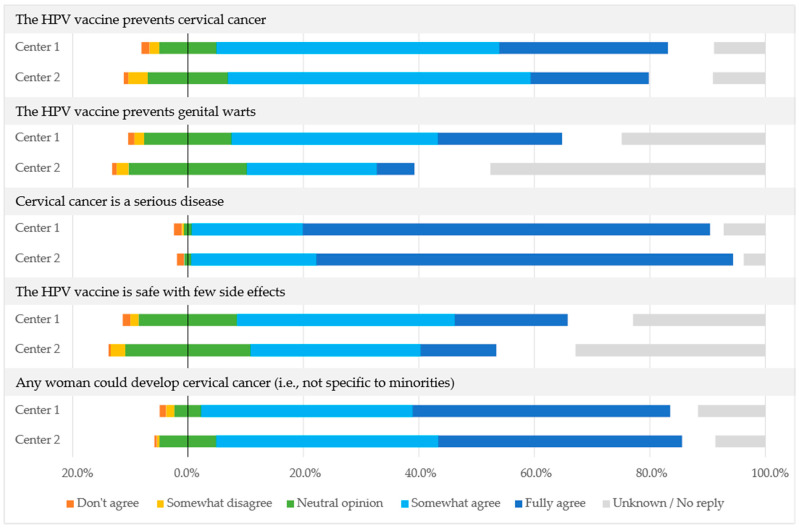
Level of agreement with the statements about the HPV vaccine and HPV-related diseases at Center 1 and Center 2, expressed as a proportion of all participants at each center.

Participants’ knowledge and opinions about the HPV vaccine were assessed using several questions in an anonymous questionnaire. Most participants that answered the question “Before reading the information provided earlier, had you ever heard about HPV vaccines?” (82.2%; 486/591) had heard of the HPV vaccine previously, with a statistically significant difference (*p* = 0.0071) between HPV vaccinated and HPV-non-vaccinated participants, with HPV-vaccinated participants more likely to have heard of the HPV vaccine previously. Figure 1 shows the level of agreement with the following statements about the HPV vaccine and HPV-related diseases: “The HPV vaccine prevents cervical cancer”; “The HPV vaccine prevents genital warts”; “Cervical cancer is a serious disease”; “The HPV vaccine is safe with few side effects”; and “Any woman could develop cervical cancer (i.e., not specific to minorities).” More than 70% (70.5%, 256/363 at Center 1 and 72.4%, 176/243 at Center 2) of participants at both centers fully agreed that cervical cancer is a serious disease. About half (50.5%, 306/606) somewhat agreed that the HPV vaccine prevents cervical cancer, and 29.2% (106/363) and 20.6% (50/243) fully agreed with this statement at Centers 1 and 2, respectively. Fewer participants (21.5% (78/363) and 6.6% (16/243)) fully agreed and 35.8% (130/363) and 22.6% (55/243) somewhat agreed at Centers 1 and 2, respectively, that the HPV vaccine also prevents genital warts.

**Figure 2:**
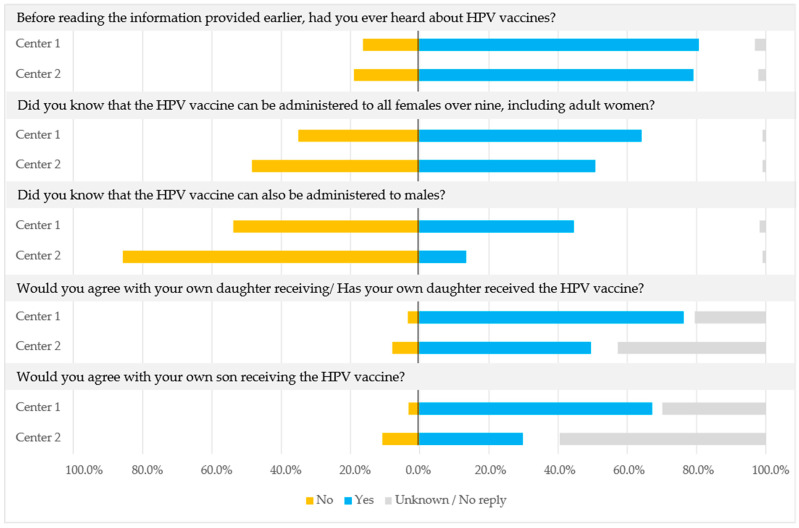
Knowledge about HPV vaccine administration and willingness to vaccinate children against HPV at Center 1 and Center 2, expressed as a proportion of all participants at each center. Participants were asked to reply to the last two questions even if they did not have children.

**Figure 3:**
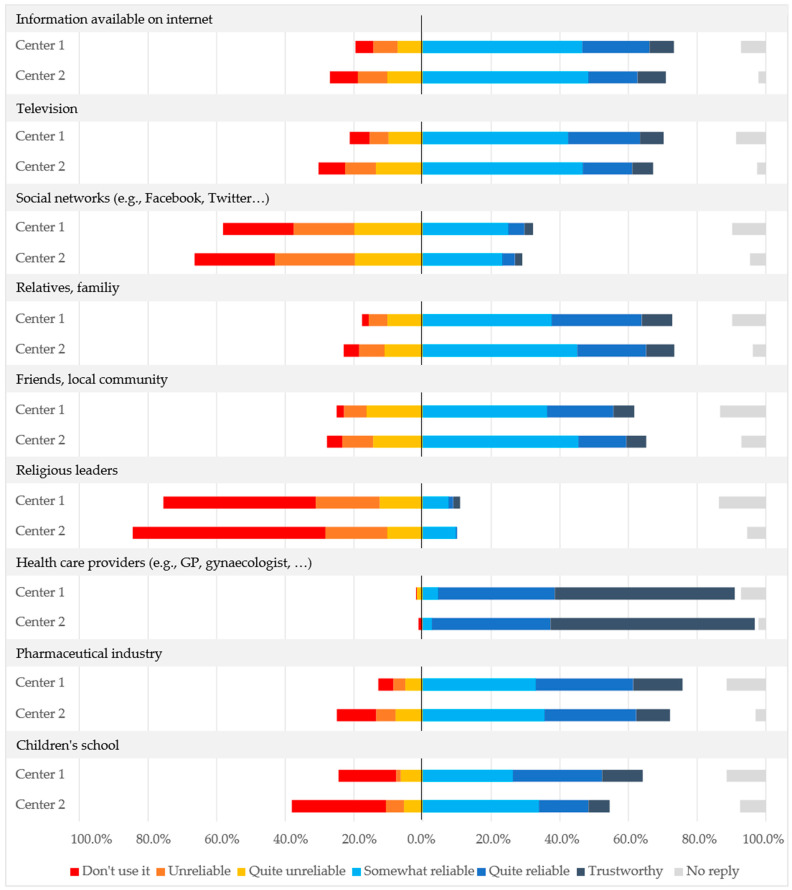
Use of and level of trust in various sources from which participants at Center 1 and Center 2 obtain medical and vaccine information, expressed as a proportion of all participants at each center.

When asked if they knew that the HPV vaccine can be administered to all females over nine years old, including adult women, 64.2% (233/363) and 50.8% (124/244) agreed at Centers 1 and 2, respectively (Figure 2). Fewer participants (44.6%, 162/363 and 13.5%, 33/244 at Centers 1 and 2, respectively) were aware of the fact that the HPV vaccine can also be administered to males, especially at Center 2. At Centers 1 and 2, 76.3% (277/363) and 49.6% (121/244) of participants would be willing to have or have had their own daughters vaccinated against HPV, and 67.2% (244/363) and 29.9% (73/244) would be willing to have their sons vaccinated against HPV. Figure 3 shows the responses to the question of where participants obtain medical information or information about vaccines and how much they trust that information. The most trusted source of information was healthcare providers (e.g., GPs or gynecologists) for 52.3% (190/363) of participants at Center 1 and 59.4% (145/244) at Center 2. Participants considered the pharmaceutical industry to be quite to somewhat reliable as an information source, whereas information available on the internet, on television, from relatives or family, from friends or the local community, and from children’s schools was considered less reliable.

**Figure 4:**
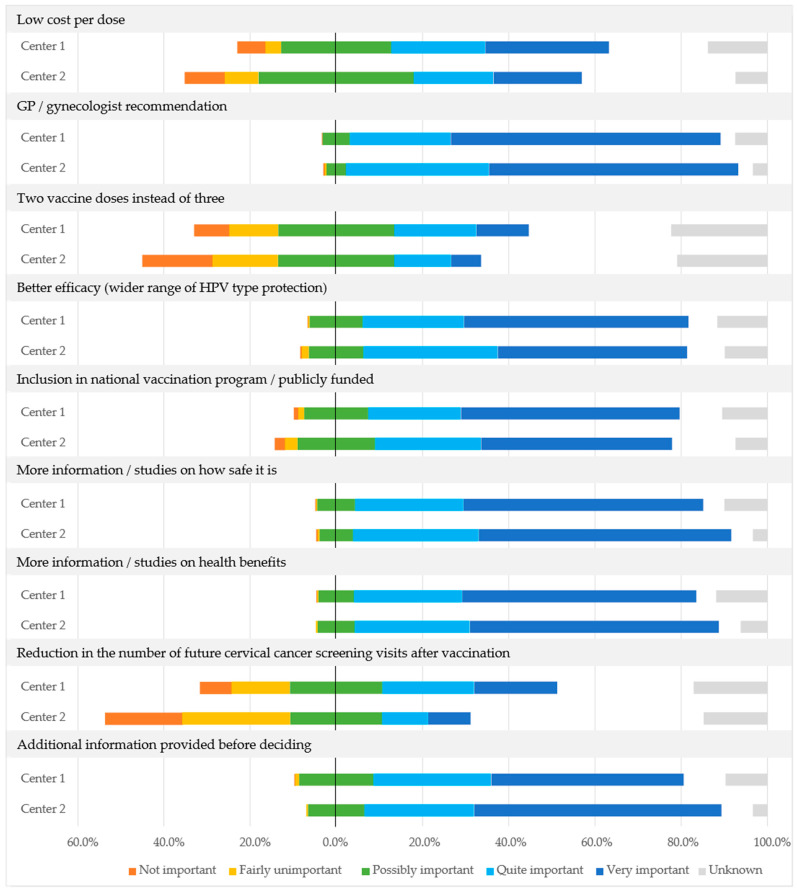
Assessment of HPV vaccine-related issues relevant to improving current HPV vaccination coverage in the Slovenian general population at Center 1 and Center 2, expressed as a proportion of all participants at each center.

The most important vaccine-related issues relevant to improving current HPV vaccination coverage in Slovenia’s general population were the following: recommendation by the primary care physician or gynecologist, need for more information or studies on vaccine safety and health benefits, additional information before making a decision, better vaccine efficacy, and inclusion in the national vaccination program or public funding for vaccination. Less relevant were lower cost per dose, two instead of three doses of vaccine, and potential reduction in the number of future cervical cancer screening visits after HPV vaccination (Figure 4).

Participants were also asked to indicate the reason(s) for accepting or refusing HPV vaccination. Most women accepted HPV vaccination in this study because they believed that the HPV vaccine protects against cervical cancer or genital warts (93.4%, 281/301), because cervical cancer is a serious disease (82.7%, 249/301), because the HPV vaccine is safe (66.8%, 201/301) or offered for free (62.8%, 189/301), and finally because it is recommended to get the vaccine (55.5%, 167/301); an additional 3.3% (10/301) stated other reasons for accepting HPV vaccination. On the other hand, the reasons for refusing HPV vaccination in this study were a need for more information (31.4%, 96/306), concerns about HPV vaccine safety or side effects (29.4%, 90/306), a need to consult other people or concerns about whether the HPV vaccine works and how long protection lasts (12.1%, 37/306), dislike of vaccines in general (11.8%, 36/306), a feel that they might not benefit from HPV vaccine protection (5.2%, 16/306), and lack of time or personal advice not to get vaccinated (1.3% each, 4/306). Nearly 6% (5.9%, 18/306) refused to answer the question, and 3.6% (11/306) stated other reasons for refusal.

The clinical part of the questionnaire completed by gynecologists recorded the cervical cytology, colposcopy, histology, and HPV results obtained during the last previous cervical cancer screening visit by the HPV-vaccinated women. Of 301 HPV-vaccinated women, 264 (87.7%) had normal cytology results, 22 (7.3%) had atypical squamous cervical cells of undetermined significance (ASC-US), six (2.0%) had a high-grade squamous intraepithelial lesion (HSIL), four (1.3%) had a low-grade squamous intraepithelial lesion (LSIL), two (0.7%) had atypical squamous cells for which HSIL (ASC-H) could not be excluded, and one (0.3%) had glandular abnormalities. In two women (0.7%), the cytology result was not obtained.

Adverse events after HPV vaccination were reported by 13.3% (40/301) of women vaccinated in this study, and a total of 45 events were reported. The most adverse events were of low intensity (95.6%; 43/45), mainly pain, swelling and redness at the injection site, headache, and fatigue, lasting from a few days to a month after vaccination. There were two reported cases of moderate adverse events (high body temperature and headache on day one; headache for three weeks), but neither the women affected required drug treatment or hospitalization.

## 4. Discussion

Vaccination against HPV as a tool for primary prevention of cervical cancer has dramatically expanded the world’s potential for the elimination of cervical cancer and most probably other HPV-related neoplasms in the anogenital and head and neck areas. In the 16 years since its introduction, HPV vaccination has seen many positive developments such as a reduced number of doses and more flexible schedules, which have reduced costs significantly and facilitated program implementation and gradual program expansion to multiple-age cohorts and both sexes [24]. All three HPV vaccines licensed in Europe are extremely safe and highly effective but still underused, especially in males, HIV-positive individuals, and adults. It is a consensus opinion in the HPV community that both sexes are responsible for HPV transmission and that both sexes should be vaccinated to share the burden in reducing the risk of HPV-related disease, as well as have equal access to direct vaccine benefits [25].

This study assessed the acceptability of HPV vaccination by adult women in Slovenia and identified determinants of uptake and completion of the vaccination schedule for the first time. Although it was performed in a relatively small country, we are persuaded that knowledge gained in this study can be reliably applied to other similar countries in central and eastern Europe, some of which have the highest burden of cervical cancer in Europe, in terms of both incidence and mortality. Despite the fact that (i) only 1.6% of participants had a history of medical problems linked with previous (non-HPV) vaccinations, (ii) more than 90% of participants thought that cervical cancer is a serious medical condition, and (iii) we offered the HPV vaccine at no cost, more than half of eligible women refused to be vaccinated against HPV. Because the market price for three-course vaccination in Slovenia (outside the national program, where it is free) ranges from EUR 180 to 300, we deemed that offering vaccination at no cost would be an important incentive for vaccine uptake, which did not turn out to be the case. However, as expected, the acceptance rate of HPV vaccination among adult women (49.6%) observed in this study mirrored the coverage rates Slovenia achieved in the national HPV vaccination program for girls attending the sixth grade of primary school in 2016–2018 (46.4%–49.5%) [26]. Although the two centers involved in the Slovenian study used the same recruitment protocol, a significant difference in HPV vaccine uptake between the two centers was observed in this study reflecting significantly different coverage rates for girls recorded across different regions in Slovenia in 2016–2018 (33.9–35.9% in the Ljubljana region versus 78.3–84.5% in the Ravne region) [26]. The observed regional difference in the vaccination uptake was not so evident in other countries that participated in the COHEAHR-WP4 study [22]. In other COHEAHR-WP4 countries that used the convenience sampling approach, the overall vaccination uptake rate was higher than in Slovenia, ranging from 77.3% to 92.6%, while in countries with a population-based approach, it was 30.3–41.8% [22]. In the COHEAHR-WP4 study, the participant’s age showed no significant impact on vaccine uptake [22]. Apart from COHEAHR-WP4, there are few data available in the peer-reviewed literature on HPV vaccine uptake by adult women in other countries, but the vaccination coverage achieved among girls usually reflects the coverage among adult women [27]. As expected, in this study, only 76.3% and 49.6% of women from Center 1 and Center 2, respectively, would have vaccinated their daughters, and an even lower proportion of women would have vaccinated their sons (67.2% at Center 1 and 29.9% at Center 2). In contrast, local initiatives in several Slovenian municipalities have proven that it is possible to substantially improve vaccination coverage among both girls and boys through the proactive engagement of medical doctors caring for school children [28].

One of the main factors when deciding to accept the vaccine or not is informed knowledge about the vaccine, which was also shown in this study because the great majority of vaccinated participants had previously heard about the HPV vaccine and many of those that refused vaccination stated that they needed more information and counseling. Interestingly, although up to 80% of women believed that the HPV vaccine prevents cervical cancer, significantly fewer at both centers were confident that the nonavalent HPV vaccine prevents genital warts. Many of the participants were also unaware that adult women and men can also be vaccinated against HPV, although the efficacy of the HPV vaccine in these cohorts has been demonstrated in large clinical trials [29,30,31]. The lack of knowledge suggests that the general population is poorly informed about the prophylactic potential of the HPV vaccine beyond cervical cancer, necessitating enhancement of dissemination of knowledge by health professionals and national HPV vaccination campaigns.

In this study, women that refused vaccination had significantly higher educational attainment, similar to other countries participating in the COHEAHR-WP4 study as well as in other studies from France, Italy, and Norway [32,33,34]. To the best of our knowledge, the opposite finding has been observed only in Denmark [35]. We can speculate that women with higher education are more skeptical about HPV vaccination, have lower trust in healthcare professionals and global health authorities, or are more actively involved in decision-making and seek information about the HPV vaccine but do not receive a convincing recommendation and/or trusted information from healthcare professionals and encounter rumors and misinformation about adverse events of the HPV vaccines that are widespread online and disseminated by social media. This is not a surprise because unfortunately, some health professionals show signs of vaccine hesitancy and negative beliefs concerning the safety of HPV vaccination [36]. Thus, there is a need to reach the widest possible range of health professionals and to ensure that their knowledge about the benefits and safety of HPV vaccination is continuously updated. Women in committed relationships and married women in this study were less inclined toward HPV vaccination most probably due to the perception of lower risk of HPV infection and HPV-related neoplasms in such partnerships, as described previously [22,37].

In addition to the lack of reliable and trusted information, the second most important factor influencing the decision to refuse vaccination in this study was safety concerns. Approximately half of the participants challenged the safety of the HPV vaccine. In addition, approximately one-tenth of participants refused vaccination because they disliked vaccines in general, although only 1.6% of participants reported that they had a history of medical problems linked with previous (non-HPV) vaccination(s). General vaccine aversion and low confidence in the safety of the HPV vaccine specifically have also been previously identified as major factors in the low uptake of the HPV vaccine [38]. However, the safety of the HPV vaccine was evaluated in large clinical trials prior to vaccine approval and is continuously being monitored in post-marketing studies worldwide. Although HPV vaccines are extremely safe and safety concerns are unfounded [39,40,41], unfortunately, reports of possible adverse events have attracted media interest and spread rumors around the world, causing confidence in the safety of HPV vaccines to wane and leading to dramatic declines in HPV vaccination coverage in some countries such as Japan, Colombia, Denmark, and Ireland [42,43]. Unfortunately, the problem of vaccine distrust became even more prominent during the SARS-CoV-2 pandemic and will very likely remain in the near future, affecting the acceptance of all vaccines, including the HPV vaccine.

Although this study was not sufficiently powered to appropriately address potential adverse events of the HPV vaccine, most of them were mild, with pain, redness, and swelling at the injection site most frequently reported. In addition, there were two reported moderate adverse events, but neither of the women required any medical treatment or hospitalization.

This study has some limitations: (i) it was conducted in only two selected centers, which may not be representative of the entire country; (ii) the recruitment of study participants in the healthcare centers may have overrepresented women with higher concerns for their health and/or women interested in free HPV vaccination may have been referred by other study participants; (iii) the limited study size.

## 5. Conclusions

This study has shown that, despite offering the HPV vaccine at no cost due to various reasons (the major being safety concerns), more than half of Slovenian women 25 to 45 years old refused to be vaccinated against HPV. To improve vaccination coverage among all people that would benefit from HPV vaccination, including adult women, it is necessary to disseminate information about the benefits and safety of HPV vaccination to the widest possible range of health professionals and to ensure that their knowledge is continuously updated. Concurrently, trusted information about HPVs, HPV-related diseases, HPV vaccines, the proven safety and efficacy of these vaccines, and the opportunity to protect adult women and men through HPV vaccination should be widely disseminated to the general public using well-planned educational campaigns and all available communication channels.

## Figures and Tables

**Table 1:** The main characteristics of the Slovenian study participants in the COHEAHR-WP4 study (*n* = 607) separated according to the decision to vaccinate against HPV or not. Values *p* ≤ 0.05 were considered statistically significant (marked in bold).

	Vaccinated		Non-vaccinated		*p*-Value
	*n* or Years	%	*n* or Years	%	
**Number of participants enrolled**					
Center 1	242	66.7	121	33.3	**<0.0001**
Center 2	59	24.2	185	75.8	
Total	301	49.6	306	50.4	
**Age (average +** ***SD*** **)**					
Center 1	35.6 ± 5.8		36.3 ± 5.4		0.2674
Center 2	35.3 ± 5.7		33.6 ± 5.8		0.5156
Total	35.5 ± 5.8		34.7 ± 5.8		0.0729
**Level of education**					
Center 1					
Up to secondary	133	70.4	56	29.6	0.1210
Above secondary	109	62.6	65	37.4	
Center 2					
Up to secondary	23	25.0	69	75.0	0.8136
Above secondary	36	23.7	116	76.3	
Total					
Up to secondary	156	55.5	125	44.5	**0.0068**
Above secondary	145	44.5	181	55.5	
**Marital status**					
Center 1					
Single/divorced	41	74.5	14	25.5	0.1593
In a stable relationship/married	191	64.7	104	35.3	
Center 2					
Single/divorced	22	44.9	27	55.1	**0.0004**
In a stable relationship/married	37	19.1	157	80.9	
Total					
Single/divorced	63	60.6	41	39.4	**0.0100**
In a stable relationship/married	228	46.6	261	53.4	
**Previous cervical cancer screening**					
Center 1					
Yes	186	65.0	100	35.0	0.2439
No	50	72.5	19	27.5	
Center 2					
Yes	54	24.1	170	75.9	0.8086
No	5	26.3	14	73.7	
Total					
Yes	240	47.1	270	52.9	**0.0077**
No	55	62.5	33	37.5	
**Regular cervical cancer screening visits**					
Center 1					
Yes	163	64.9	88	35.1	0.8300
No	17	63.0	10	37.0	
Center 2					
Yes	51	23.9	162	76.1	0.5290
No	3	33.3	6	66.7	
Total					
Yes	214	46.1	250	53.9	0.2817
No	20	55.6	16	44.4	
**Lifetime cervical cancer screening visits**					
Center 1					
≤2	8	61.5	5	38.5	0.7543
>2	153	65.7	80	34.3	
Center 2					
≤2	2	66.7	1	33.3	0.1514
>2	47	22.9	158	77.1	
Total					
≤2	10	62.5	6	37.5	0.1986
>2	200	45.7	238	54.3	

Abbreviations: *n* = number; *SD* = standard deviation.

## Data Availability

The data used during the current study are available from the corresponding author upon reasonable request.

## Supplementary Materials

The following supporting information can be downloaded at https://www.mdpi.com/article/10.3390/vaccines11020423/s1: Supplement S1: Study questionnaire; Table S2: Level of agreement with statements about the HPV vaccine and HPV-related diseases at Center 1 and Center 2, expressed as a proportion of all participants at each center; Table S3: Knowledge about HPV vaccine administration and willingness to vaccinate children against HPV at Center 1 and Center 2, expressed as a proportion of all participants at each center. Participants were asked to reply to the last two questions even if they do not have children; Table S4: Use of and level of trust in various sources from which participants at Center 1 and Center 2 obtain medical and vaccine information, expressed as a proportion of all participants at each center; Table S5: Assessment of HPV vaccine-related issues relevant to improving current HPV vaccination coverage in the Slovenian general population at Center 1 and Center 2, expressed as a proportion of all participants at each center.
