# Supplementary material for: Assessment of Acceptability and Determinants of Uptake and Schedule Completion of Human Papillomavirus (HPV) Vaccine by 25 to 45 Years Old Women in Slovenia
Source: Vaccines (Basel). 2023 Feb 12;11(2):423. doi: 10.3390/vaccines11020423 (PMC9964155; doi:10.3390/vaccines11020423)
Supplement: Supplementary file 1 [file vaccines-11-00423-s001.zip › vaccines-2194757-SI.pdf]

SUPPLEMENT

Assessment of acceptability and determinants of uptake and schedule completion of human papillomavirus (HPV) vaccine by 25 to 45 years old women in Slovenia

S1: Study questionnaire

Study ID

		----			----				
Country			Site			Patient number			

This section is to be completed by your study doctor.

BACKGROUND INFORMATION

In the following questions we will ask you about your demographic data, some medical history, and your attitude toward cervical cancer screening.

1. Date the survey is completed: → ___________

2. Your date of birth: → ___________

3. Your country of birth: → ___________

4. Your maximum education level completed:

☐ None completed / did not attend school

☐ Primary

☐ Secondary / high school

☐ University

☐ Other. Please specify:________________

5. Could you indicate the number of years spent in education? ___________

6. What is your current marital status?

☐ Single/divorced

☐ In a stable relationship / married

☐ Widowed

☐ Prefer not to disclose my marital status

7. Have you ever been diagnosed by a doctor with one of these conditions? Please tick all that apply.

☐ Asthma. If yes, please provide approximate year of diagnosis: I__I__I

☐ Anogenital warts. If yes, please provide approximate year of diagnosis: I__I__I

☐ Cancer. If yes, please:

- Specify where: ____________________________ and
- Provide approximate year of diagnosis: I__I__I

☐ Allergy. If yes, please:

- Specify to what: ____________________________ and
- Provide approximate year of diagnosis: I__I__I

☐ Hepatitis. If yes, please provide approximate year of diagnosis: I__I__I

☐ Disease of the uterus. If yes, please:

- Specify which: ______________________________ and
- Provide approximate year of diagnosis: I__I__I

8. You are attending a screening clinic for the prevention of cervical cancer. Do you know which test is being used by your gynecologist?

☐ Yes, cervical cytology or PAP test.

This test analyses a sample of your cervix obtained using a brush, and it looks for the presence of pre-cancerous lesions on it.

☐ Yes, a test for HPV.

This test analyses a sample of your cervix obtained using a brush, and it looks for the presence of the HPV virus on it.

☐ Yes, none of the above. Please specify: ______________________

☐ No, I do not know (even after the explanations above).

9. Apart from the present visit, have you ever had a screening test before (PAP / cervical cytology or smear / HPV test) for the prevention of cervical cancer?

☐ No previous test (please continue at question 12)

☐ Yes. Can you remember approximately when this test was performed? ☐ Less than 1 year ago

☐ 2–3 years ago

☐ 4 or more years ago

☐ I do not remember

10. If yes to question 9, are you a regular participant in the cervical screening program? (defined as performing a cervical examination every three years)

☐ Yes (please continue at question 11)

☐ No. Could you provide us with a reason among the options below?

☐ Lack of time
☐ Discomfort with the procedure

☐ Misinformation or lack of information on the need for periodic cervical screening

☐ Economic reasons (including work leave or travel costs)

☐ Other. Please specify: _________________

11. Can you estimate the number of screening visits you have attended in your lifetime?

☐ 1 visit

☐ 2–5 visits

☐ More than 5 visits

☐ Unknown

12. Have you ever had any medical problem with any previous vaccine?

☐ No

☐ Yes. Can you specify which medical problem and which vaccine?

____

13. Have you ever refused to receive a vaccine?

☐ No ☐ Yes. Which vaccine? ______________________

KNOWLEDGE ABOUT THE HPV VACCINE

The HPV vaccine is currently recommended to be administered to women over nine years old and to boys as a strategy to prevent cervical and ano-genital lesions. In Slovenia, the current HPV vaccination program vaccinates women 11 to 12 years old.

14. Before reading the information provided earlier, had you ever heard about HPV vaccines?

☐ No ☐ Yes

15. Please indicate your current level of agreement with the following questions on the HPV vaccine and HPV-related diseases. (check the corresponding box)

	Don't agree	Somewhat disagree	Neutral opinion	Somewhat agree	Fully agree	Unknown / No reply
The HPV vaccine prevents cervical cancer						

The HPV vaccine prevents genital warts						
Cervical cancer is a serious disease						
The HPV vaccine is safe with few side effects						
Any woman could develop cervical cancer (i.e., not specific to minorities)						

16. Did you know that the HPV vaccine can be administered to all females over nine, including adult women?

☐ No ☐ Yes

17. Did you know that the HPV vaccine can also be administered to males?

☐ No ☐ Yes

18. Would you agree with your own daughter receiving / Has your own daughter received the HPV vaccine? (Please reply even if you don't have children).

☐ No ☐ Yes ☐ Unknown

19. Would you agree with your own son receiving the HPV vaccine? (Please reply even if you don't have children).

☐ No ☐ Yes ☐ Unknown

20. How do you usually find medical information or information on vaccines, and how much do you trust their information?

Below there is a list of common sources of information. Please express your opinion about the quality of information provided and your trust in it.

	Don't use it	Unreliable	Quite unreliable	Somewhat reliable	Quite reliable	Trustworthy
Information available on internet						
Television						
Social networks (e.g., Facebook, Twitter)						
Relatives, family						
Friends, local community						
Religious leaders						
Healthcare providers (e.g., GP, gynecologist)						

Pharmaceutical industry						
Children's school						

THE ACCEPTABILITY OF THE HPV VACCINE

We would like to know your opinion on some topics related to the HPV vaccine in order to improve the current HPV vaccination coverage in the general population.

Please rate the following topics based on relevance to get the population vaccinated

	Not important	Fairly unimportant	Possibly relevant	Quite important	Very important	Unknown
Low cost per dose						
GP/gynecologist recommendation						
Two vaccine doses instead of three						
Better efficacy (wider range of HPV type protection)						
Inclusion in national vaccination program / publicly funded						
More information / studies on how safe it is						
More information / studies on health benefits						
Reduction in the number of future cervical cancer screening visits after vaccination						
Additional information provided before deciding						

Finally, we would like you to consider the second part of this study, on whether you would accept an invitation to be vaccinated with the HPV vaccine. If you accept, this would be given to you free of charge, but you would need to come for three visits (to receive the three HPV vaccine doses) within a year.

Before you make any decision, you can re-read the attached information, discuss it with your gynecologist, family doctor, or friends and family, or phone or e-mail the principal investigator (Dr. Mario Poljak, MD), and he will answer your questions.

A. **If you accept the HPV vaccine invitation,** you will be asked to sign the appropriate informed consent form. Your gynecologist will take a cervical smear that will be tested for HPV and/or precancerous lesions. You and your gynecologist will schedule the first dose of the vaccine. You will receive the second dose of the HPV vaccine two months after the first dose. The third dose of the HPV vaccine is given six months after the first

dose. Depending on the results of screening for cervical cancer or the presence of high-risk HPV, your gynecologist may decide to change the vaccination schedule.

Please choose the reasons why you would like to take up this offer of HPV vaccination: (check all applicable)

☐ The HPV vaccine is safe

☐ Vaccine protects against cervical cancer / genital warts

☐ The HPV vaccine is offered for free

☐ It is recommended to get the HPV vaccine

☐ Cervical cancer is a serious disease

☐ All of the above ☐ Other: ____

B. **If you refuse the HPV vaccine invitation,** you can decide not to reply to the following question (check the option below). However, your answer will be much appreciated to better understand the general population's opinion on the HPV vaccine. Independently of your reasons, we will not try to convince you to get vaccinated.

Please choose the main reasons for refusal of HPV vaccination: (check all applicable)

☐ I refuse to reply this question

☐ I am concerned about HPV vaccine safety / side effects

☐ I am concerned about whether the HPV vaccine works and how long it lasts

☐ I do not like vaccines

☐ I do not have time (e.g., work schedule, transportation issues)

☐ I feel I might not benefit from the HPV vaccine protection

☐ I have been personally advised not to get vaccinated

☐ I need more information

☐ I need to consult other people

☐ Other: ____

You have been provided with a patient information sheet. In this document you can find the contact details of the researchers involved in the study. Feel free to contact them if you change your mind regarding accepting the HPV vaccination.

THANK YOU VERY MUCH FOR YOUR ANSWERS

These will help us better understand people's appreciation of the HPV vaccine.

Table S2. Level of agreement with statements about the HPV vaccine and HPV-related diseases at Center 1 and Center 2, expressed as a proportion of all participants at each center.

	Don't agree % (*n*/*N*)	Somewhat disagree % (*n*/*N*)	Neutral opinion % (*n*/*N*)	Somewhat agree % (*n*/*N*)	Fully agree % (*n*/*N*)	Unknown / No reply % (*n*/*N*)
The HPV vaccine prevents cervical cancer						
Center 1	1.4 (5/363)	1.7 (6/363)	9.9 (36/363)	49.0 (178/363)	29.2 (106/363)	8.8 (32/363)
Center 2	0.8 (2/244)	3.3 (8/244)	13.9 (34/244)	52.5 (128/244)	20.5 (50/244)	9.0 (22/244)
Total	1.2 (7/607)	2.3 (14/607)	11.5 (70/607)	50.4 (306/607)	25.7 (156/607)	8.9 (54/607)
The HPV vaccine prevents genital warts						
Center 1	1.1 (4/363)	1.7 (6/363)	15.2 (55/363)	35.8 (130/363)	21.5 (78/363)	24.8 (90/363)
Center 2	0.8 (2/244)	2.0 (5/244)	20.5 (50/244)	22.5 (55/244)	6.6 (16/244)	47.5 (116/244)
Total	1.0 (6/607)	1.8 (11/607)	17.3 (105/607)	30.5 (185/607)	15.5 (94/607)	33.9 (206/607)
Cervical cancer is a serious disease						
Center 1	1.4 (5/363)	0.3 (1/363)	1.4 (5/363)	19.3 (70/363)	70.5 (256/363)	7.2 (26/363)
Center 2	1.2 (3/244)	0.0 (0/244)	1.2 (3/244)	21.7 (53/244)	72.1 (176/244)	3.7 (9/244)
Total	1.3 (8/607)	0.2 (1/607)	1.3 (8/607)	20.3 (123/607)	71.2 (432/607)	5.8 (35/607)
The HPV vaccine is safe with few side effects						
Center 1	1.4 (5/363)	1.4 (5/363)	17.1 (62/363)	37.7 (137/363)	19.6 (71/363)	22.9 (83/363)
Center 2	0.4 (1/244)	2.5 (6/244)	21.7 (53/244)	29.5 (72/244)	13.1 (32/244)	32.8 (80/244)
Total	1.0 (6/607)	1.8 (11/607)	18.9 (115/607)	34.4 (209/607)	17.0 (103/607)	26.9 (163/607)
Any woman could develop cervical cancer (i.e., not specific to minorities)						
Center 1	1.1 (4/363)	1.4 (5/363)	4.7 (17/363)	36.6 (133/363)	44.6 (162/363)	11.6 (42/363)
Center 2	0.4 (1/244)	0.4 (1/244)	9.8 (24/244)	38.5 (94/244)	42.2 (103/244)	8.6 (21/244)
Total	0.8 (5/607)	1.0 (6/607)	6.8 (41/607)	37.4 (227/607)	43.7 (265/607)	10.4 (63/607)

Table S3. Knowledge about HPV vaccine administration and willingness to vaccinate children against HPV at Center 1 and Center 2, expressed as a proportion of all participants at each center. Participants were asked to reply to the last two questions even if they do not have children.

	No<br>% (*n*/*N*)	Yes<br>% (*n*/*N*)	Unknown / No reply<br>% (*n*/*N*)
Before reading the information provided earlier, had you ever heard about HPV vaccines?			
Center 1	16.3 (59/363)	80.7 (293/363)	3.0 (11/363)
Center 2	18.9 (46/244)	79.1 (193/244)	2.0 (5/244)
Total	17.3 (105/607)	80.1 (486/607)	2.6 (16/607)
Did you know that the HPV vaccine can be administered to all females over nine, including adult women?			
Center 1	35.0 (127/363)	64.2 (233/363)	0.8 (3/363)
Center 2	48.4 (118/244)	50.8 (124/244)	0.8 (2/244)
Total	40.4 (245/607)	58.8 (357/607)	0.8 (5/607)
Did you know that the HPV vaccine can also be administered to males?			
Center 1	53.7 (195/363)	44.6 (162/363)	1.7 (6/363)
Center 2	85.7 (209/244)	13.5 (33/244)	0.8 (2/244)
Total	66.6 (404/607)	32.1 (195/607)	1.3 (8/607)
Would you agree with your own daughter receiving / Has your own daughter received the HPV vaccine?			
Center 1	3.3 (12/363)	76.3 (277/363)	20.4 (74/363)
Center 2	7.8 (19/244)	49.6 (121/244)	42.6 (104/244)
Total	5.1 (31/607)	65.6 (398/607)	29.3 (178/607)
Would you agree with your own son receiving the HPV vaccine?			
Center 1	3.0 (11/363)	67.2 (244/363)	29.8 (108/363)
Center 2	10.7 (26/244)	29.9 (73/244)	59.4 (145/244)
Total	6.1 (37/607)	52.2 (317/607)	41.7 (253/607)

Table S4. Use of and level of trust in various sources from which participants at Center 1 and Center 2 obtain medical and vaccine information, expressed as a proportion of all participants at each center.

	Don't use it	Unreliable	Quite unreliable	Somewhat reliable	Quite reliable	Trustworthy	No reply
Information available on internet							
Center 1	5.2 (19/363)	7.2 (26/363)	7.2 (26/363)	46.6 (169/363)	19.6 (71/363)	7.2 (26/363)	7.2 (26/363)
Center 2	8.2 (20/244)	8.6 (21/244)	10.2 (25/244)	48.4 (118/244)	14.3 (35/244)	8.2 (20/244)	2.0 (5/244)
Total	6.4 (39/607)	7.7 (47/607)	8.4 (51/607)	47.3 (287/607)	17.5 (106/607)	7.6 (46/607)	5.1 (31/607)
Television							
Center 1	5.8 (21/363)	5.5 (20/363)	9.9 (36/363)	42.4 (154/363)	20.9 (76/363)	6.9 (25/363)	8.5 (31/363)
Center 2	7.8 (19/244)	9.0 (22/244)	13.5 (33/244)	46.7 (114/244)	14.3 (35/244)	6.1 (15/244)	2.5 (6/244)
Total	6.6 (40/607)	6.9 (42/607)	11.4 (69/607)	44.2 (268/607)	18.3 (111/607)	6.6 (40/607)	6.1 (37/607)
Social networks (e.g., Facebook, Twitter)							
Center 1	20.7 (75/363)	17.6 (64/363)	19.8 (72/363)	25.1 (91/363)	4.7 (17/363)	2.5 (9/363)	9.6 (35/363)
Center 2	23.4 (57/244)	23.4 (57/244)	19.7 (48/244)	23.4 (57/244)	3.7 (9/244)	2.0 (5/244)	4.5 (11/244)
Total	21.7 (132/607)	19.9(121/607)	19.8(120/607)	24.4 (148/607)	4.3 (26/607)	2.3 (14/607)	7.6 (46/607)
Relatives, family							
Center 1	1.9 (7/363)	5.5 (20/363)	10.2 (37/363)	37.7 (137/363)	26.2 (95/363)	8.8 (32/363)	9.6 (35/363)
Center 2	4.5 (11/244)	7.4 (18/244)	11.1 (27/244)	45.1 (110/244)	20.1 (49/244)	8.2 (20/244)	3.7 (9/244)
Total	3.0 (18/607)	6.3 (38/607)	10.5 (64/607)	(247/607)	40.7 (/607)	8.6 (52/607)	7.2 (44/607)
Friends, local community							
Center 1	2.2 (8/363)	6.6 (24/363)	16.3 (59/363)	36.4 (132/363)	19.3 (70/363)	6.1 (22/363)	13.2 (48/363)
Center 2	4.5 (11/244)	9.0 (22/244)	14.3 (35/244)	45.5 (111/244)	13.9 (34/244)	5.7 (14/244)	7.0 (17/244)
Total	3.1 (19/607)	7.6 (46/607)	15.5 (94/607)	40.0 (243/607)	17.1 (104/607)	5.9 (36/607)	10.7 (65/607)
Religious leaders							
Center 1	44.4 (161/363)	18.7 (68/363)	12.4 (45/363)	7.7 (28/363)	1.4 (5/363)	1.9 (7/363)	13.5 (49/363)
Center 2	56.1 (137/244)	18.0 (44/244)	10.2 (25/244)	9.8 (24/244)	0.4 (1/244)	0.0 (0/244)	5.3 (13/244)
Total	49.1 (298/607)	18.5(112/607)	11.5 (70/607)	8.6 (52/607)	1.0 (6/607)	1.2 (7/607)	10.2 (62/607)
Healthcare providers (e.g., GP, gynecologist)							
Center 1	0.3 (1/363)	0.0 (0/363)	1.7 (6/363)	4.7 (17/363)	33.9 (123/363)	52.3 (190/363)	7.2 (26/363)
Center 2	0.8 (2/244)	0.4 (1/244)	0.0 (0/244)	2.9 (7/244)	34.4 (84/244)	59.4 (145/244)	2.0 (5/244)
Total	0.5 (3/607)	0.2 (1/607)	1.0 (6/607)	4.0 (24/607)	34.1 (207/607)	55.2 (335/607)	5.1 (31/607)
Pharmaceutical industry							
Center 1	4.4 (16/363)	3.6 (13/363)	5.0 (18/363)	33.1 (120/363)	28.4 (103/363)	14.3 (52/363)	11.3 (41/363)
Center 2	11.5 (28/244)	5.7 (14/244)	7.8 (19/244)	35.7 (87/244)	26.6 (65/244)	9.8 (24/244)	2.9 (7/244)
Total	7.2 (44/607)	4.4 (27/607)	6.1 (37/607)	34.1 (207/607)	27.7 (168/607)	12.5 (76/607)	7.9 (48/607)
Children’s school							
Center 1	16.8 (61/363)	1.4 (5/363)	6.3 (23/363)	26.4 (96/363)	25.9 (94/363)	11.8 (43/363)	11.3 (41/363)
Center 2	27.5 (67/244)	5.3 (13/244)	5.3 (13/244)	34.0 (83/244)	14.3 (35/244)	6.1 (15/244)	7.4 (18/244)
Total	21.1 (128/607)	3.0 (18/607)	5.9 (36/607)	29.5 (179/607)	21.3 (129/607)	9.6 (58/607)	9.7 (59/607)

Table S5. Assessment of HPV vaccine-related issues relevant to improving current HPV vaccination coverage in the Slovenian general population at Center 1 and Center 2, expressed as a proportion of all participants at each center.

	Not important	Fairly unimportant	Possibly important	Quite important	Very important	Unknown
Low cost per dose						
Center 1	6.6 (24/363)	3.6 (13/363)	25.6 (93/363)	21.8 (79/363)	28.7 (104/363)	13.8 (50/363)
Center 2	9.4 (23/244)	7.8 (19/244)	36.1 (88/244)	18.4 (45/244)	20.5 (50/244)	7.4 (18/244)
Total	7.7 (47/607)	5.3 (32/607)	29.8 (181/607)	20.4 (124/607)	25.4 (154/607)	11.2 (68/607)
GP/gynecologist recommendation						
Center 1	0.3 (1/363)	0.0 (0/363)	6.3 (23/363)	23.4 (85/363)	62.5 (227/363)	7.4 (27/363)
Center 2	0.4 (1/244)	0.4 (1/244)	4.5 (11/244)	33.2 (81/244)	57.8 (141/244)	3.3 (8/244)
Total	0.3 (2/607)	0.2 (1/607)	5.6 (34/607)	27.3 (166/607)	60.6 (368/607)	5.8 (35/607)
Two vaccine doses instead of three						
Center 1	8.3 (30/363)	11.3 (41/363)	27.0 (98/363)	19.0 (69/363)	12.1 (44/363)	22.3 (81/363)
Center 2	16.4 (40/244)	15.2 (37/244)	27.0 (66/244)	13.1 (32/244)	7.0 (17/244)	20.9 (51/244)
Total	11.5 (70/607)	12.9 (78/607)	27.0 (164/607)	16.6 (101/607)	10.0 (61/607)	21.7 (132/607)
Better efficacy (wider range of HPV type protection)						
Center 1	0.3 (1/363)	0.3 (1/363)	12.4 (45/363)	23.4 (85/363)	52.1 (189/363)	11.6 (42/363)
Center 2	0.4 (1/244)	1.6 (4/244)	12.7 (31/244)	31.1 (76/244)	43.9 (107/244)	9.8 (24/244)
Total	0.3 (2/607)	0.8 (5/607)	12.5 (76/607)	26.5 (161/607)	48.8 (296/607)	10.9 (66/607)
Inclusion in national vaccination program / publicly funded						
Center 1	1.1 (4/363)	1.4 (5/363)	14.9 (54/363)	21.5 (78/363)	50.7 (184/363)	10.5 (38/363)
Center 2	2.5 (6/244)	2.9 (7/244)	18.0 (44/244)	24.6 (60/244)	44.3 (108/244)	7.4 (18/244)
Total	1.6 (10/607)	2.0 (12/607)	16.1 (98/607)	22.7 (138/607)	48.1 (292/607)	9.2 (56/607)
More information / studies on how safe it is						
Center 1	0.3 (1/363)	0.3 (1/363)	8.8 (32/363)	25.1 (91/363)	55.6 (202/363)	9.9 (36/363)
Center 2	0.4 (1/244)	0.4 (1/244)	7.8 (19/244)	29.1 (71/244)	58.6 (143/244)	3.3 (8/244)
Total	0.3 (2/607)	0.3 (2/607)	8.4 (51/607)	26.7 (162/607)	56.8 (345/607)	7.2 (44/607)
More information / studies on health benefits						
Center 1	0.3 (1/363)	0.3 (1/363)	8.3 (30/363)	25.1 (91/363)	54.3 (197/363)	11.8 (43/363)
Center 2	0.0 (0/244)	0.4 (1/244)	8.6 (21/244)	26.6 (65/244)	57.8 (141/244)	6.1 (15/244)
Total	0.2 (1/607)	0.3 (2/607)	8.4 (51/607)	25.7 (156/607)	55.7 (338/607)	9.6 (58/607)
Reduction in the number of future cervical cancer screening visits after vaccination						
Center 1	7.4 (27/363)	13.5 (49/363)	21.5 (78/363)	21.2 (77/363)	19.3 (70/363)	17.1 (62/363)
Center 2	18.0 (44/244)	25.0 (61/244)	21.3 (52/244)	10.7 (26/244)	9.8 (24/244)	14.8 (36/244)
Total	11.7 (71/607)	18.1 (110/607)	21.4 (130/607)	17.0 (103/607)	15.5 (94/607)	16.1 (98/607)
Additional information provided before deciding						
Center 1	0.3 (1/363)	0.8 (3/363)	17.4 (63/363)	27.3 (99/363)	44.6 (162/363)	9.6 (35/363)
Center 2	0.0 (0/244)	0.4 (1/244)	13.1 (32/244)	25.4 (62/244)	57.4 (140/244)	3.3 (8/244)
Total	0.2 (1/607)	0.7 (4/607)	15.7 (95/607)	26.5 (161/607)	49.8 (302/607)	7.1 (43/607)
